# Toward Noninvasive High‐Resolution In Vivo pH Mapping in Brain Tumors by ^31^P‐Informed deepCEST MRI

**DOI:** 10.1002/nbm.70060

**Published:** 2025-05-15

**Authors:** Jan‐Rüdiger Schüre, Junaid Rajput, Manoj Shrestha, Ralf Deichmann, Elke Hattingen, Andreas Maier, Armin M. Nagel, Arnd Dörfler, Eike Steidl, Moritz Zaiss

**Affiliations:** ^1^ Institute of Neuroradiology University Hospital Erlangen, Friedrich‐Alexander‐Universität Erlangen‐Nürnberg (FAU) Germany; ^2^ Institute of Neuroradiology Goethe University Hospital Frankfurt, Goethe University Frankfurt am Main Germany; ^3^ Cooperative Brain Imaging Center (CoBIC) Goethe University Frankfurt Frankfurt am Main Germany; ^4^ Department Artificial Intelligence in Biomedical Engineering Friedrich‐Alexander‐Universität Erlangen‐Nürnberg Erlangen Germany; ^5^ Institute of Radiology University Hospital Erlangen, Friedrich‐Alexander‐Universität Erlangen‐Nürnberg (FAU) Erlangen Germany; ^6^ Division of Medical Physics in Radiology German Cancer Research Center (DKFZ) Heidelberg Germany

**Keywords:** ^31^P‐MRS, AI, APTw, brain tumor, CEST, deep learning, intracellular pH, pHi

## Abstract

The intracellular pH (pH_i_) is critical for understanding various pathologies, including brain tumors. While conventional pH_i_ measurement through ^31^P‐MRS suffers from low spatial resolution and long scan times, ^1^H‐based APT‐CEST imaging offers higher resolution with shorter scan times. This study aims to directly predict ^31^P‐pH_i_ maps from CEST data by using a fully connected neuronal network. Fifteen tumor patients were scanned on a 3‐T Siemens PRISMA scanner and received ^1^H‐based CEST and T1 measurement, as well as ^31^P‐MRS. A neural network was trained voxel‐wise on CEST and T1 data to predict ^31^P‐pH_i_ values, using data from 11 patients for training and 4 for testing. The predicted pH_i_ maps were additionally down‐sampled to the original the ^31^P‐pH_i_ resolution, to be able to calculate the RMSE and analyze the correlation, while higher resolved predictions were compared with conventional CEST metrics. The results demonstrated a general correspondence between the predicted deepCEST pH_i_ maps and the measured ^31^P‐pH_i_ in test patients. However, slight discrepancies were also observed, with a RMSE of 0.04 pH units in tumor regions. High‐resolution predictions revealed tumor heterogeneity and features not visible in conventional CEST data, suggesting the model captures unique pH information and is not simply a T1 segmentation. The deepCEST pH_i_ neural network enables the APT‐CEST hidden pH‐sensitivity and offers pH_i_ maps with higher spatial resolution in shorter scan time compared with ^31^P‐MRS. Although this approach is constrained by the limitations of the acquired data, it can be extended with additional CEST features for future studies, thereby offering a promising approach for 3D pH imaging in a clinical environment.

## Graphical abstract

^31^P‐MRS is a noninvasive method for measuring intracellular pH. This study presents a ^31^P‐informed deep CEST approach for generating high resolved pHi data by using proton‐based APT‐CEST input data. The higher resolution enables contrast features, which are not a pure T1 segmentation and differ from conventional CEST metrics

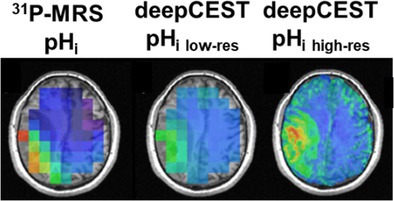

**Abbreviations**
^31^P‐MRS: phosphorus magnetic resonance spectroscopy
AACID: amide–amine concentration‐independent detection
APT: amide proton transfer
AREX: apparent exchange dependent relaxation
CEST: chemical exchange saturation transfer
FoV: field of view
GLLN: Gaussian negative log‐likelihood loss
GRE: gradient echo
MTR_asym_
: magnetization transfer ratio asymmetry
pHi: intracellular pH
PVE: partial volume effect
qT1: quantitative T1
ReLu: rectified linear unit
RMSE: root mean square error
SSIM: structure similarity index measure

## 1 Introduction

The measurement of the intracellular pH (pH_i_) value in the human brain provides the opportunity to assess physiological information that is of interest in different diseases [1, 2]. Especially in malignant brain tumors, pH_i_ alterations are common due to the Warburg effect [3]. Such information provides further insight into metabolic activities, tumor progression or even resistance to therapy [4].

The conventional method for noninvasive pH_i_ detection is phosphorus MR spectroscopy (^31^P‐MRS), which can be traced back to the work of Moon and Richards, who investigated intracellular phosphates and their varying chemical shifts due to different protonation states [5]. The most sensitive signal toward pH‐changes is the inorganic phosphate (Pi). By taking its spectral distance from the signal of phosphocreatine (PCr) and translation into the modified Henderson–Hasselbalch equation, it is possible to calculate the pH_i_ [6]. Nevertheless, ^31^P‐MRS exhibits a low spatial resolution (e.g., 1–3 cm^3^), long scan times due to the low signal‐to‐noise ratio (SNR), required measurement repetitions or use of spatial encoding techniques such as chemical shift imaging. In addition, special hardware is required for planning and measuring phosphorus data, which involves changing to a phosphorus coil. All these factors hamper ^31^P‐MRS availability in the clinical routine.

An alternative ^1^H‐based approach to noninvasively access pH_i_ and to overcome ^31^P‐MRS limitations is the usage of Chemical Exchange Saturation Transfer (CEST) imaging via the indirect detection of the acid‐/base‐catalyzed chemical exchange rate between labile protons from mobile molecules with water protons. Already in its first description in vivo amide‐proton‐transfer (APT) CEST imaging promised pH‐weighting via the so called magnetization transfer ratio asymmetry (MTR_asym_) [7, 8]. Through maintaining the high SNR of proton MRI it could provide the advantage of a higher spatial resolution and scan times below 2 min [9]. Such a 3D pH imaging is traded as the biggest promise or the holy grail of CEST MRI and highly suited for noninvasive characterization of different pathologies.

Although the chemical exchange rate of the amide protons has been demonstrated to be a reliable method to determine pH changes under experimental conditions [10, 11, 12, 13], it is also possible to achieve pH‐dependent image contrast by using the exchange rates of the amine [14, 15] or hydroxyl groups [16]. However, considerable changes arise in its application in vivo. In particular, pathologies like brain tumors lead to widespread changes in the tissue composition. Previous research has introduced further corrections methods to counteract spillover and interfering MT effects on the CEST signal and its isolation from other overlying CEST pools and different longitudinal relaxation times [17]. However, ^1^H‐APT‐CEST imaging for pH mapping in brain tumors has not demonstrated a clear spatial correlation when compared with ^31^P‐pH_i_ maps. Although the MTR_asym_ and the isolated APT signal according to the Lorentzian difference correlated well with phosphorus pH_i_ in brain tumors [18], the correlation disappears with additional T1 correction in pathologically altered tissue types [19]. Consequently, additional corrections may be needed to account for different amide proton concentrations or temperature fluctuations [20], which impact the exchange rate and thus the CEST contrast. These factors likely explain why conventional approaches have not yet been able to accurately predict pH in brain tumors so far.

Deep learning approaches, on the other hand, are known for their performance in ill‐posed situations and could overcome these problems. In this preliminary study, we present a novel approach for directly learning ^31^P‐pH_i_ maps from APT‐CEST data using a voxel‐wise deep neural network‐based CEST (deepCEST) approach.

## 2 Methods

### 2.1 Data Acquisition

Data from a previous study [19] were reanalyzed, summarizing 15 patients with brain tumors and 1 healthy subject. All subjects provided informed consent in accordance with ethical guidelines and were scanned on a 3‐T whole‐body MRI system (MAGNETOM Prisma, Siemens Healthineers, Erlangen, Germany). APT‐CEST and quantitative T1 (qT1) measurements were performed with a 20‐channel phase‐array head/neck ^1^H receive coil, while for the acquisition of ^31^P data, a double‐tuned ^1^H/^31^P head coil (Rapid Biomedical, Rimpar, Germany) was used.

For qT1 measurement, two 3D GRE datasets were acquired within 9:48 min according to the variable flip angle (VFA) method [21] with FA_1_ = 4°, FA_2_ = 24°, isotropic voxel size of 1 mm, FoV = 256 × 224 × 160 mm^3^, TE/TR = 6.7 ms/16.4 ms, bandwidth = 222 Hz/Pixel. In order to correct for B_1_ inhomogeneity, two additional GRE datasets with and without magnetization preparation via a 45° RF pulse were acquired within 1:45 min according to Volz et al. [22], while for B_0_ correction a 2D multislice dual‐echo GRE sequence was performed (1:03 min) and used with exported magnitude and phase information.

For APT‐CEST, we used a fast multislice CEST‐EPI sequence [23] with a voxel size of 3 × 3 × 4 mm^3^ across 16 slices, FoV = 256 × 256 mm^2^, bandwidth = 2298 Hz/pixel, TE1/TE2 = 22.08 ms/23.28 ms for Dixon correction and a TR = 8000 ms. The saturation module consisted of an initial train of 10 rectangular pulses to approach steady‐state, followed by single rectangular pulses (duration = 250 ms, spacing td = 250 ms, B_1_ = 1 μT), to maintain saturation. CEST data were acquired in the spectral range from +8 to −8 ppm (increment 0.5 ppm), while the spectral range of interest—from ± 3 to ± 4 ppm—was sampled higher (increment 0.1 ppm). The total acquisition time was 16:48 min.

Phosphorus spectroscopic data were acquired in 15 min by using a 3D CSI with a voxel size of 30 × 30 × 25 mm^3^, FA = 60°, FoV = 240 × 240 × 200 mm^3^, bandwidth = 2000 Hz/pixel, TR/TE = 2000 ms /2.3 ms, and 10‐fold averaging. The matrix size was extrapolated from 8 × 8 × 8 to 16 × 16 × 16 by k‐space zero filling. An anatomical reference scan was further included for planning the spectroscopic measurement. Therefore, a 3D T1‐w MPRAGE sequence was used with 1 mm isotropic resolution, FoV = 256 × 256 × 192 mm^2^, TI/TR/TE = 900 ms/1900 ms/3.2 ms.

### 2.2 Postprocessing

First, CEST data were motion corrected using mc‐flirt [24] from the FMRIB Software Library (FSL) to align all images. The motion parameters (rotation and translation) were checked to ensure that the displacements remain within the voxel size, thus confirming the adequacy of the motion correction. After alignment, voxel‐wise Z‐spectra were calculated by normalizing with an unsaturated reference scan and correcting for B_0_ inhomogeneities according to WASSR [25]. The resulting Z‐spectra were used to calculate the MTR_asym_ according to the literature [7]. To generate qT1 maps, the two GRE datasets were processed using the VFA method and corrected for insufficient RF‐spoiling [21]. In detail, the B_1_ maps were used to increase the accuracy of the VFA analysis by calculating the actual flip angles for each pixel. Afterwards co‐registration was performed onto the unsaturated CEST dataset. For generating ground‐truth pH maps from ^31^P‐MRS, raw data were first registered to the anatomical MPRAGE dataset employing an in‐house Matlab script, which allows reslicing and the selection of several voxels on an MRSI grid overlay. The spectra were then fitted in jMRUI with the nonlinear least square fitting algorithm AMARES [26], before the spectral distance between Pi and PCr was translated into the modified Henderson–Hasselbalch equations [6]. The calculation of the intracellular pH was achieved by utilizing the dissociation constant of dihydrogen phosphate (${\mathrm{pK}}_{a}$ = 6.77) and its chemical shift ($\delta \;H_{2}{\mathrm{PO}}_{4}^{-}$ = 3.29 ppm) as well as the chemical shift of hydrogen phosphate ($\delta {\mathrm{HPO}}_{4}$ = 5.68 ppm) were used in the brain [18, 27].

A prediction model was developed in Pytorch, utilizing a feedforward fully connected network incorporating four fully connected layers. The network's architecture comprises an input layer, which receives a high‐dimensional vector summarizing voxel‐wise and normalized data. Besides the B_0_‐corrected Z‐spectrum, the vector contains values from the higher sampled APTw MTR_asym_ between +3 and +4 ppm as well as a quantitatively measured T1 value for the T1‐dependence of water within the amide proton transfer rate (APTR) [7].

- B_0_‐corrected Z‐spectra (including 51 offsets, −8 to 8 ppm)
- APT‐weighted MTR_asym_ (3:0.1:4 ppm)
- Normalized qT1 value

The input data were trained voxel‐wise on co‐registered and resliced ^31^P‐pH_i_ maps from data of 11 brain tumor patients. To exclude outliers in ^31^P‐MRS data near the skull, input and target data were multiplied with an eroded brain mask, resulting in a total number of 107,786 CEST spectra and pH values. 80% of the data were used for training, while the remaining 20% were used for validating the model. Input and target data were standardized by calculating mean and standard deviation.

**FIGURE 1:**
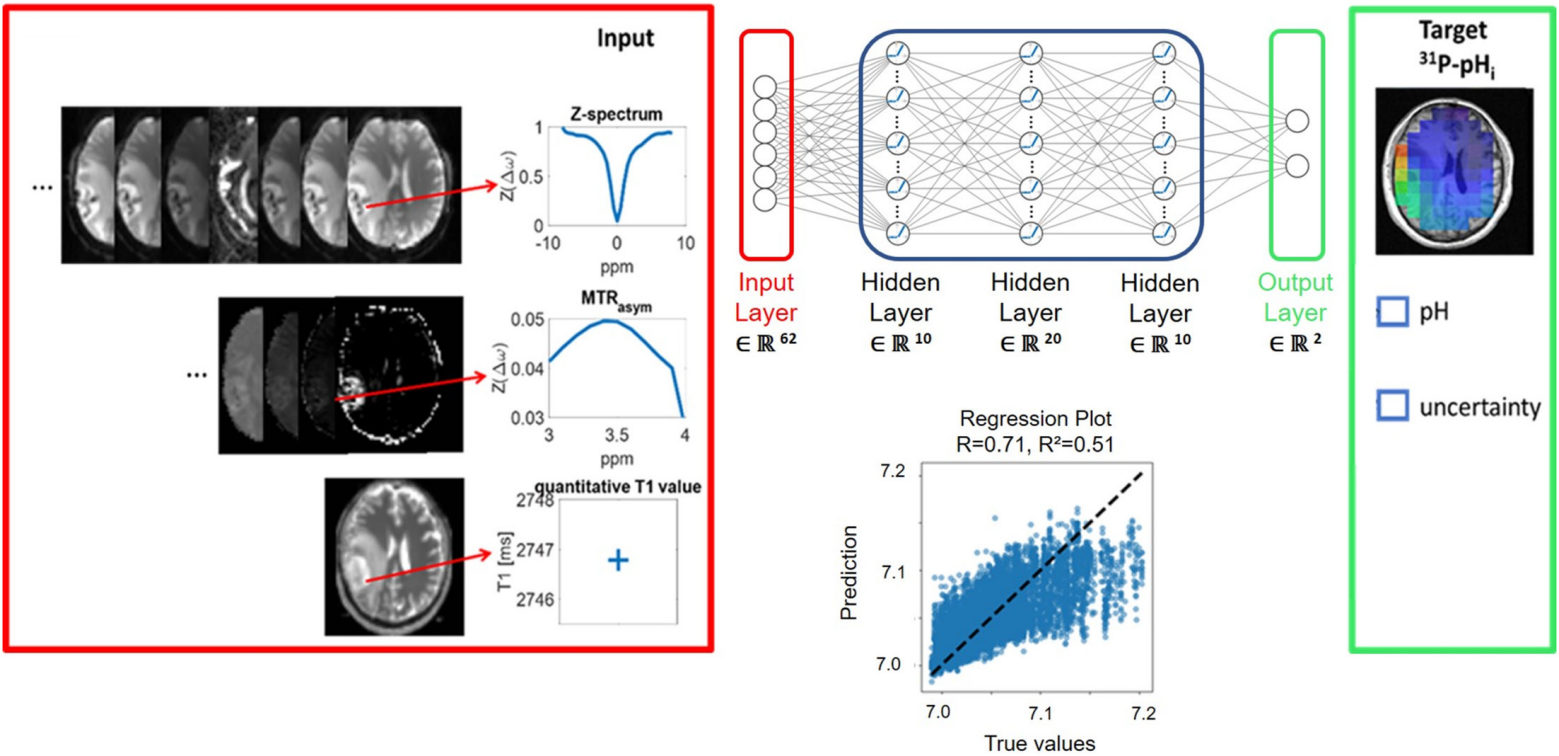
Illustration of the predictive model, which incorporates input (Z‐spectrum, MTR_asym_, qT1) and target (^31^P‐pH_i_) data. The convergence of the deepCEST‐pH_i_ training process after 1000 iterations showed a good performance for training and test data with a RMSE below 0.04 pH_i_ units, while the regression plot revealed a positive correlation between the ^31^P target data and the deepCEST‐pH_i_ prediction with 51% of the validation data explained.

After an initial network architecture comprising four hidden layers with 64, 128, 256, and 512 neurons respectively, the network showed signs of overfitting. We therefore reduced the architecture to three layers with 10, 20, and 10 neurons respectively (see Supporting Information S1 and S2). Each layer incorporates a Rectified Linear Unit (ReLU) activation function. These were followed by a probabilistic output layer that provides the mean and uncertainty prediction value [28]. The network was trained with a Gaussian negative log‐likelihood loss (GNLL) function, which enables the uncertainty estimation. Optimization was achieved by using the Adam optimizer, which utilizes adaptive learning rates and moment estimation in order to handle noisy and large‐scale problems. Further training parameters are given by a learning rate of 0.001, batch size of 256 and 1000 epochs. Figure 1 illustrates the structure of the fully connected network, comprising three input (red) and one target (green) datasets. After training, the test dataset revealed a good correlation between prediction and training values as shown in the regression plot (*r* = 0.71, *R* ^2^ = 0.51). The deepCEST pH_i_ training process demonstrates convergence across 1000 iterations, exhibiting a high level of performance for both training and test data, with a root mean square error (RMSE) below 0.04 pH_i_ units.

The ^31^P‐informed predictive model was evaluated on four brain tumor patients and one healthy subject, which were not included in the training process. In order to facilitate a comparison between the predicted and original ^31^P‐pH_i_ maps, the deepCEST pH_i_ maps were resliced and co‐registered back onto the high‐resolution MPRAGE, which was used for planning the spectroscopic image acquisition. To address the different spatial resolution, the higher resolved deepCEST pH_i_ maps were additionally down‐sampled to the same resolution as the original phosphorus pH_i_ maps. To exclude the possibility that the prediction is mainly based on higher T1 values in the tumor region rather than the CEST data, we additionally attacked the predictive model by adding false T1 values resembling a tumor in the healthy white matter of the healthy volunteer.

Statistical analysis was performed by calculating the structural similarity (SSIM), RMSE, coefficient of determination, and Pearson correlation and using Bland–Altman analysis.

## 3 Results

**FIGURE 2:**
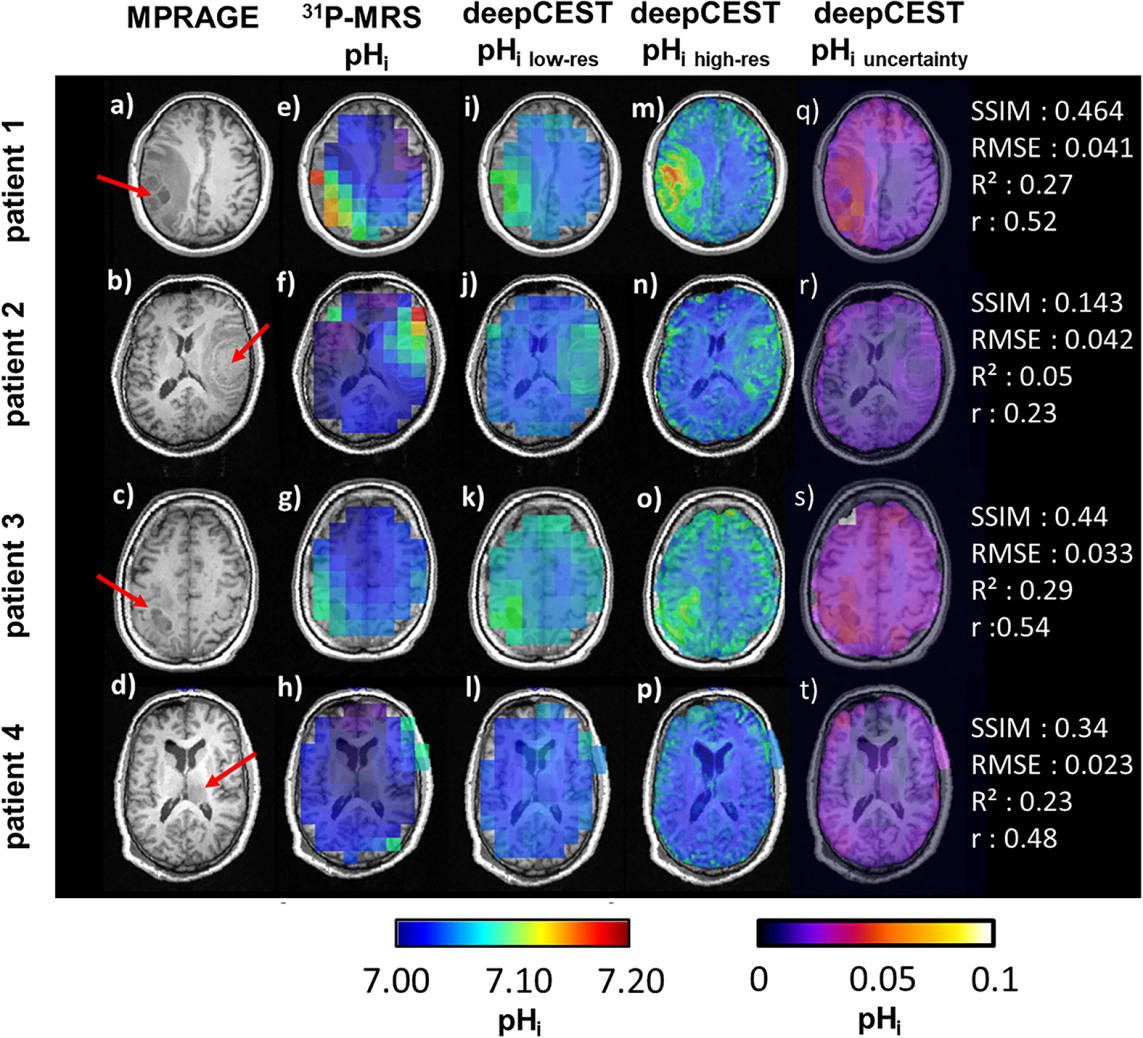
The following unseen test datasets are presented, each showing a representative slice from four patients with brain tumors, marked by the red arrows on the MPRAGE dataset (a–d). The different tumor types are as follows: (1) glioblastoma (IDH‐wt), (2) metastasis (malignant melanoma), (3) glioblastoma (IDH‐wt), and (4) glioblastoma (IDH‐wt). Corresponding intracellular pH (pH_i_) maps (e–h) indicate higher pH_i_ in the tumor region with exception of patient 4 in the left ventricular structure (h). Down‐sampled deepCEST pH_i_ predictions to the same spatial resolution as the ^31^P‐data reveal a similar image contrast with higher pH_i_ values in the tumor region (i–l), while the higher resolved predictions even show more highlighted pH with new hot spots compared with the original ^31^P‐pH_i_ (m–p). The variation of pH_i_ given by its uncertainty differs between the patients and is in general below 0.05 pH units, which agrees with the observed RMSE during training from Figure 1. A statistical analysis between ^31^P‐pH_i_ and down‐sampled deepCEST is provided for the chosen slice in the right‐hand column.

Figure 2 compares spectroscopic and deepCEST pH_i_ images of the four unseen patient datasets shown by their MPRAGE (a–d). Observing ^31^P‐data, elevated pH_i_ can be detected across the tumor regions, while the values decrease toward the contralateral hemisphere (e–h). Down‐sampled deepCEST pH_i_ maps (i–l) show a good similarity toward the measured ^31^P‐pH_i_. Nevertheless, there were slight discrepancies between the localization of higher pH_i_ values in tumor regions in the phosphorus data compared with the down‐sampled deepCEST pH_i_ predictions. The uncertainty maps show higher deviations for Patients 1 and 3 (~0.05 pH units) in the tumor region, while lower deviations were detected for Patients 2 and 4 (~0.02 pH units). The RMSE, on the other hand, showed comparable results for all four patients. Further, the predictions in contralateral white matter also differs from the ground‐truth data, as the deepCEST provides slightly elevated pH_i_ values. Overall, this discrepancy seems to be reflected in the SSIM as well as the correlation coefficient. When observing the higher resolved deepCEST data (m‐p), the pH_i_ maps even reveals new substructures that are not visible in the ^31^P‐pH maps nor in the down‐sampled predictions, which could indicate pH_i_ changes within the strong tumor heterogeneity. Interestingly, Subject 4 does not show elevated pH values in the tumor region neither in ^31^P‐data nor in both deepCEST pH_i_ maps.

**FIGURE 3:**
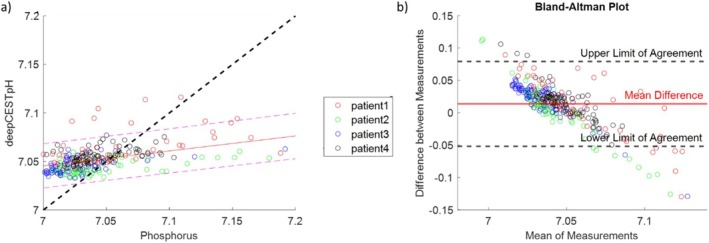
Comparison of the down‐sampled deepCEST pH_i_ and phosphorus pH demonstrates in (a) the correlation within one representative tumor slice for each patient. The corresponding Bland–Altman plot is shown in (b) and suggest a bias of 0.01 between both methods, while lower pH values seem overestimated and higher pH values underestimated. The different tumor types for each patient are as follows: (1) glioblastoma (IDH‐wt), (2) metastasis (malignant melanoma), (3) glioblastoma (IDH‐wt), and (4) glioblastoma (IDH‐wt).

The analysis of the down‐sampled predictions versus the ground truth data from all patients is shown in Figure 3a within a representative slice yielding an RMSE of 0.036 and a standard error of ± 0.01. These findings further show that the predicted pH_i_ values fall within the 95% confidence interval of the measured ^31^P‐pH_i_ values, but only a small part of the variance is explained by them (*r* = 0.45 and *R* ^2^ = 0.2). Patient 1 exhibits some outliers, as the predicted pH_i_ value was higher in certain voxels in the tumor area as well as in the contralateral white matter. This is also shown in the corresponding Bland–Altman plot (Figure 3b), which exhibits a mean difference of 0.01 ± 0.03, which corroborates a small bias. The data further indicate a notable overestimation for lower pH_i_ values, whereas in the tumor region, there is an underestimation. The lower and upper limits of agreement are given by +0.075 and −0.05.

**FIGURE 4:**
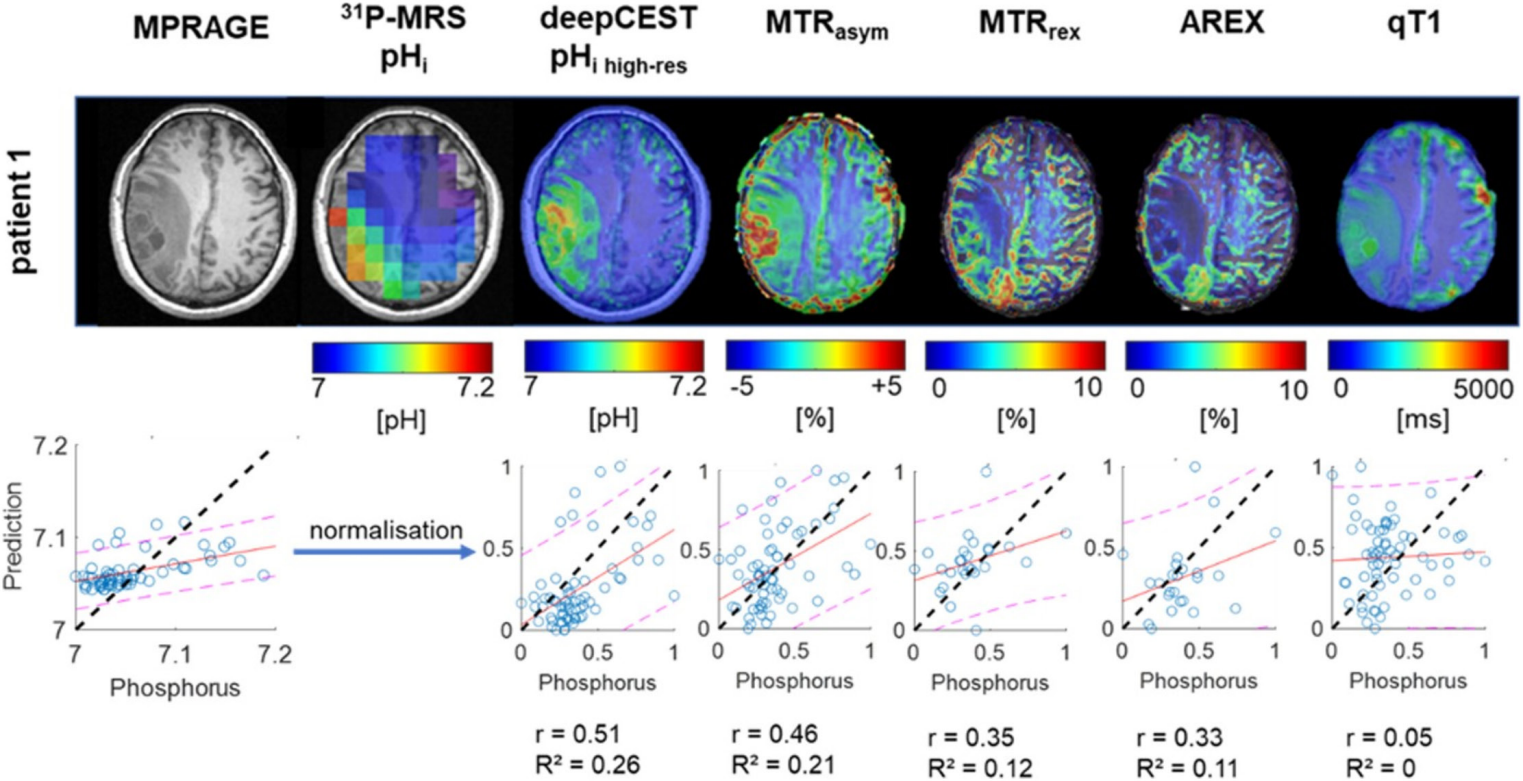
A comparison of the high‐resolution deepCEST pHi prediction with the various amide CEST metrics at Δ*ω* = 3.5 ppm (MTR_asym_, MTR_rex_, AREX) and qT1 data set is presented. Down‐sampled and normalized data were used for correlation plots. It is observed that deepCEST pHi exhibits the best correlation for the shown slice and further provides a distinctive distribution of image contrast within the tumor area, while the contralateral side remains stable in its image contrast.

Visual examination of the higher resolved deepCEST predictions revealed the existence of previously hidden structures, which had remained obscured due to the lower spatial resolution of the phosphorus data. To investigate if deepCEST has a valuable context and better performance than simpler and more precise CEST metrics, we compared its predictions with conventional pH‐sensitive CEST approaches, as shown in Figure 4. As demonstrated, the deepCEST prediction differs in its image contrast from other pH‐sensitive amide CEST metrics. While elevated pH_i_ values were observed with deepCEST in the tumor region surrounding the necrotic core and its periphery, the pH‐weighted MTRasym reveals a hotspot within the necrotic core. The strong image contrast is getting more reduced by the observation of the more accurate CEST metrics, which isolate the exchange‐dependent relaxation rate (Rex) such as the spillover corrected magnetization transfer ratio (MTRrex) and the further T1 corrected apparent exchange dependent relaxation (AREX). Assessing its voxel‐wise sensitivity by using the down‐sampled and normalized data toward the ground‐truth pHi data seems to conform this observation, as the data reveal an improved correlation between the deepCEST and ^31^P pH_i_ values.

**FIGURE 5:**
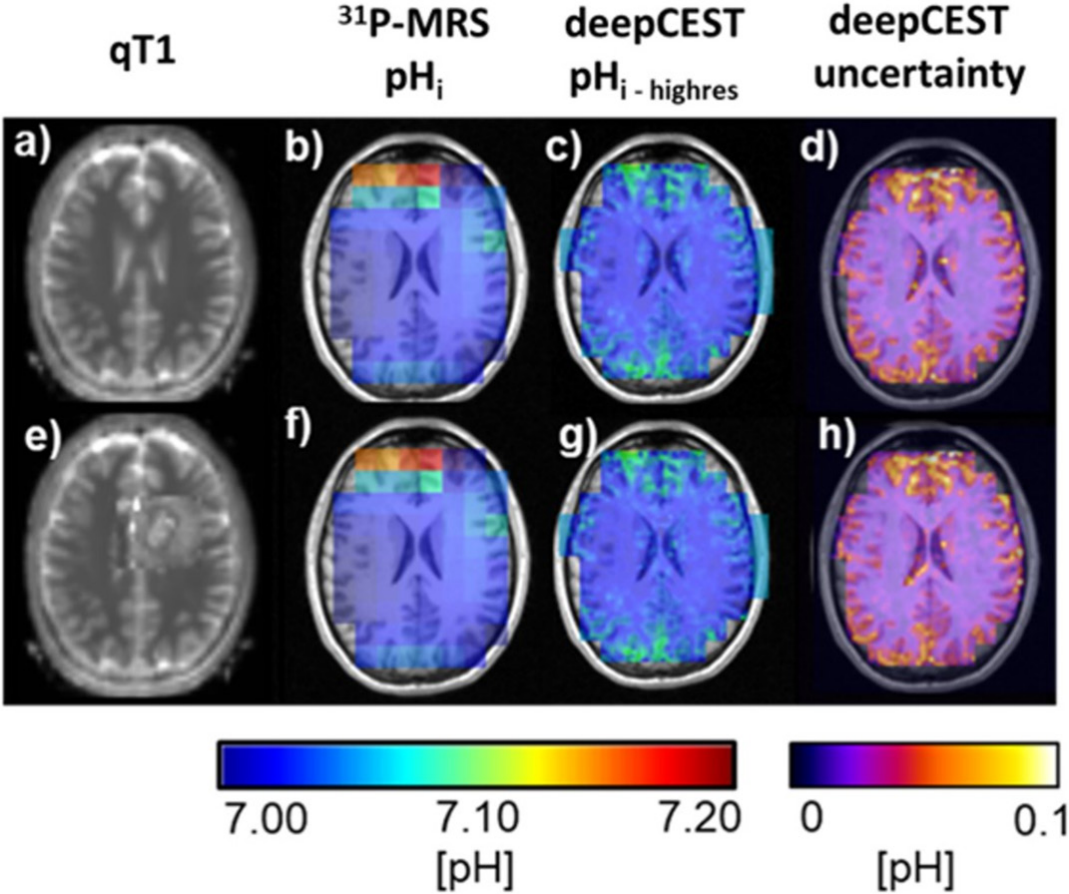
Data of healthy volunteer with corresponding qT1 map (a), original ^31^P‐pH_i_ map (b), highly resolved deepCEST prediction (c), and the predicted uncertainty (d). We now attack the predictive model by modifying qT1 data by adding a tumor into the healthy white matter (e). However, the deepCEST prediction (g) and its corresponding uncertainty (h) stay almost unchanged, indicating that the prediction is more based on the CEST input data.

In order to demonstrate that the predictive model does not simply perform a T1 segmentation and that the pH_i_ information originates mainly from the CEST data, the network was presented two different versions of the healthy subject T1 dataset: (i) the original and (ii) a modified version by manually adding a tumor within the left hemisphere (Figure 5). When observing phosphorus pH_i_, it is noticeable that the healthy subject has higher pH values in the frontal lobe, which is likely due to suboptimal B_0_ shimming, which leads to a signal distortion in the nasal region. However, when comparing the original and modified predictions in the modified tumor area, there is no significant change in the higher resolved pH_i_ nor in the provided uncertainty maps.

## 4 Discussion

The prediction of pH_i_ based on ^31^P‐informed deep CEST appears to be promising, as it has the highest correlation coefficient of all tested metrics, although it is not yet fully developed. This is reflected in the mismatch of some voxels between the ground truth data and the down‐sampled predictions, with a large deviation of 0.05 pH units in tumor tissue and a relatively stable deviation of 0.02 pH_i_ units in white matter. One of the main reasons for this deviation in some voxels origins through the limited spatial resolution of the 3D phosphorus datasets and its partial volume effects, which cause signal mixing between different tissue compartments, such as between white and grey matter (see also Figure S3). In addition, the coarse sampling of k‐space reduces the spatial encoding fidelity, leading to a broadened point‐spread function (PSF), which causes signal leakage from neighboring voxels into actual analyzed voxel. Transferring this problem onto tumor tissue with a strong tissue heterogeneity, causes a mix of signals (i) in the tumor itself, including a mixing of intra‐ and extracellular pH and (ii) with leakage from the outside voxels, probably reducing the intracellular pH contrast. This could explain the reduced pH values in the tumor areas of the deepCEST pH maps as well as the slightly increased pH values in the area of the cortical GM/CSF as seen in Figure 2 from the four patients and the healthy subject in Figure 5.

Further findings indicate that the predictive model is slightly overestimating pH_i_ values in normal appearing white matter, while underestimating tumor pH_i_ as shown in the Bland–Altman plot. This could be due to the lack of healthy subjects because the model learns just on patient data, which might already have slightly increased pH_i_ values in normal appearing white matter [4, 18], leading to a general overestimation. In tumor tissue, on the other hand, the underestimated pH_i_ values were found to have the greatest uncertainty in their prediction, as well as in their deviation from ground truth. Further, it remains unclear, if the deviation is also attributed to a change in protein/peptide concentration, which the model learned from the CEST input data. Although the heterogeneity of the tumor and the low spatial resolution of the training data make a more accurate prediction of the model difficult, an average RMSE of 0.04 pH was achieved. It is therefore all the more astonishing that the model achieves this prediction and the associated errors with trained CEST data that has only been acquired at only one B_1_ level of 1 μT. This restricts the scope of the information hidden in the Z‐spectra and corresponding MTR_asym_. Nevertheless, the low B_1_ level could lead to stronger labeling of the amide signal, which in turn could have a positive effect on the quality of the data, as recent studies at 3 T suggest [29, 30].

However, in case of Subject 4, no elevated pH was measured by ^31^P‐MRS and predicted through the model. Although this fact further strengthens the general feasibility of such a model, one could say the model more relies on T1 values. Therefore, we further illustrated in attacking the model that increased T1 values do not lead to a higher pH_i_ prediction. This was additionally checked by presenting only T1 input data to the model. However, such a prediction did not work and did not show an increase in pH_i_. When comparing the down‐sampled and normalized data toward each other, we showed that deepCEST pH_i_ and MTR_asym_ correlate strongest with phosphorus pH_i_, while other metrics such as MTR_rex_ and AREX experience a lower compliance.

One potential explanation for the inferior performance of the correlation and coefficient of determination is the inherent limitation of the target data, characterized by a lower spatial resolution, as mentioned above. Through down‐sampling of the deepCEST images, local variations of the CEST or T1 signals, which contain important information about the tissue changes, are averaged out. In the cases of MTR_rex_ and AREX, which provide a better isolation of the pH‐dependent R_ex_ term [17], the observed decline in correlation may also be attributed to their enhanced sensitivity—MTR_rex_ to T1 changes and AREX to variations in the proton fraction [19]. The high variability of both parameters in tumor tissue may further contribute to the correlation between deepCEST and phosphorus pH. Moreover, because the Henderson–Hasselbalch equation describes a logarithmic function, a linear adjustment may not be appropriate enough in the physiological range between 7 and 7.2 pH units.

Nevertheless, we demonstrate the general feasibility of predicting pH_i_ maps using a learning‐based ^31^P‐informed approach. This preliminary work on predicting intracellular pH based on a neuronal network has the major advantage of overcoming the limitations of ^31^P‐MRS by not requiring the use of special hardware such as a phosphorus coil, once the network training has been completed. Further such an approach provides higher SNR and spatial resolution by the factor of 625, when considering the voxel volumes (^31^P‐MRS: 22.500 mm^3^, ^1^H‐CEST: 36 mm^3^). This in general allows a more accurate mapping of pH changes, helping to assess disease, monitor progression and detect local variations in future studies.

The deepCEST neuronal network was chosen to contain only three hidden layers with a small number of neurons to prevent overfitting. This will change in the future, when more data are available for training. The training was performed with GNLL to model heteroscedastic uncertainties in addition to predicting pH_i_ values [28]. The performance of the network can also be improved by choosing a deep learning based Lorentzian model [31] to isolate various CEST effects instead of the MTR_asym_.

Despite the good agreement in this first attempt of predicting pH by using ^31^P‐informed deepCEST with the fully connected network, the quality of the input and target data can be further improved. One way is to acquire data at increased field strength, which offers several advantages. For example, ^31^P‐MRS gains more SNR, better‐resolved spectra, and increased spatial resolution, which improve the target data for the predictive model [20, 32, 33]. Thus, better‐resolved data also reduce the influence of partial‐volume effects (PVE) in the case of strong tumor heterogeneities. In addition, CEST imaging also profits from higher field strength due to longer T1 relaxation and the opportunity to acquire slow, medium and fast exchanging CEST regimes. One way is the usage of comprehensive CEST protocols as described in the previous work of Fabian et al. [34]. Such an additional acquisition of CEST data at different B_1_ levels would be advantageous in determining further pH‐dependent exchange rates, including those from amines and hydroxyl groups. Furthermore, it would enable the calculation of CEST ratios that are independent of their concentration.

Another approach used for pH mapping via the amide protons in iobitridol with B_1_ = 1.5 and 3 μT, which covers the physiological pH range and has been successfully tested on the phantom and animal model study [13]. To further overcome the problem of tumor heterogeneity and thus different concentrations, which might hamper adequate prediction, the utilization of amide–amine concentration‐independent detection (AACID) [35, 36] could serve to reinforce the input data. An alternative approach is the utilization of ratio maps [9] as demonstrated in a previous study at 9.4 T [12]. However, this method requires the use of porcine brain lysate for calibration. In contrast, the predictive model approach could eliminate the need for lysate and allow independent analysis of pH_i_.

While detailed studies of causality and used CEST features are required, this preliminary result is highly promising for a noninvasive 3D and high‐resolution MRI‐based pH mapping method, which could provide valuable information for brain tumor diagnosis and therapy, as well as for other diseases.

## 5 Conclusion

High‐resolution CEST–MRI–based pH_i_ mapping in brain tumors is in principle possible at 3 T using the deepCEST‐pH_i_ neural network informed by ^31^P target data. As performance is expected to increase with further added features and field strength, this guides a direction for a potential noninvasive and high‐resolution 3D pH_i_ mapping.

## Supporting information


**Figure S1** *Illustration of the training and validation loss curves according to the trained networks by using different a different number of layers and neurons.*
**Figure S2**: Comparison of the model performance and the impact of T1 in addition to figure 5 by (i) providing the model just CEST data and (ii) with additional T1. The results reveal almost similar predictions but a slight improved correspondence when T1 is also addressed.
**Figure S3:** Fitted ^31^P‐spectra from the brain, acquired at 3 T and postprocessed with jMRUI and the embedded tool AMARES (Advanced Method for accurate, robust, and efficient spectral fitting). When observing the spectra in WM (a) and WM/GM boundaries (b), both spectra seem similar, indicating the impact of WM due to PVE. When comparing WM and tumor tissue, the ^31^P‐spectra reveal a minor increase of the fitted spectral distance. However, in tumor tissue this might be also hampered due PVE.

## Data Availability

The data that support the findings of this study are available on request from the corresponding author. The data are not publicly available due to privacy or ethical restrictions.
