# Supplementary material for: Toward Noninvasive High‐Resolution In Vivo pH Mapping in Brain Tumors by 31P‐Informed deepCEST MRI
Source: NMR Biomed. 2025 May 15;38(6):e70060. doi: 10.1002/nbm.70060 (PMC12081166; doi:10.1002/nbm.70060)
Supplement: Supplementary file 1 — Figure S1 Illustration of the training and validation loss curves according to the trained networks by using different a different number of layers and neurons. Figure S2: Comparison of the model performance and the impact of T1 in addition to figure 5 by (i) providing the model just CEST data and (ii) with additional T1. The results reveal almost similar predictions but a slight improved correspondence when T1 is also addressed. Figure S3: Fitted 31P‐spectra from the brain, acquired at 3 T and postprocessed with jMRUI and the embedded tool AMARES (Advanced Method for accurate, robust, and efficient spectral fitting). When observing the spectra in WM (a) and WM/GM boundaries (b), both spectra seem similar, indicating the impact of WM due to PVE. When comparing WM and tumor tissue, the 31P‐spectra reveal a minor increase of the fitted spectral distance. However, in tumor tissue this might be also hampered due PVE. [file NBM-38-e70060-s001.docx]

**Supplementary material**

The following figures illustrate the training and validation loss curves for a variety of tested architectures comprising different numbers of neurons in their 3-layers (a to d) and an additional 4-layer (e) architecture. Although the 4-layer network [64, 128, 256, 512] can learn the training set almost perfectly, it overfits poorly - its validation loss remains higher and starts to creep up as training progresses. In contrast, the smaller three-layer models (especially [10, 20, 10] or [10, 128, 10]) exhibit more moderate gaps between training and validation. Although they do not drive the training loss towards zero, they do not increase the validation loss as much. Empirically, this indicates a better generalization for the task at hand.


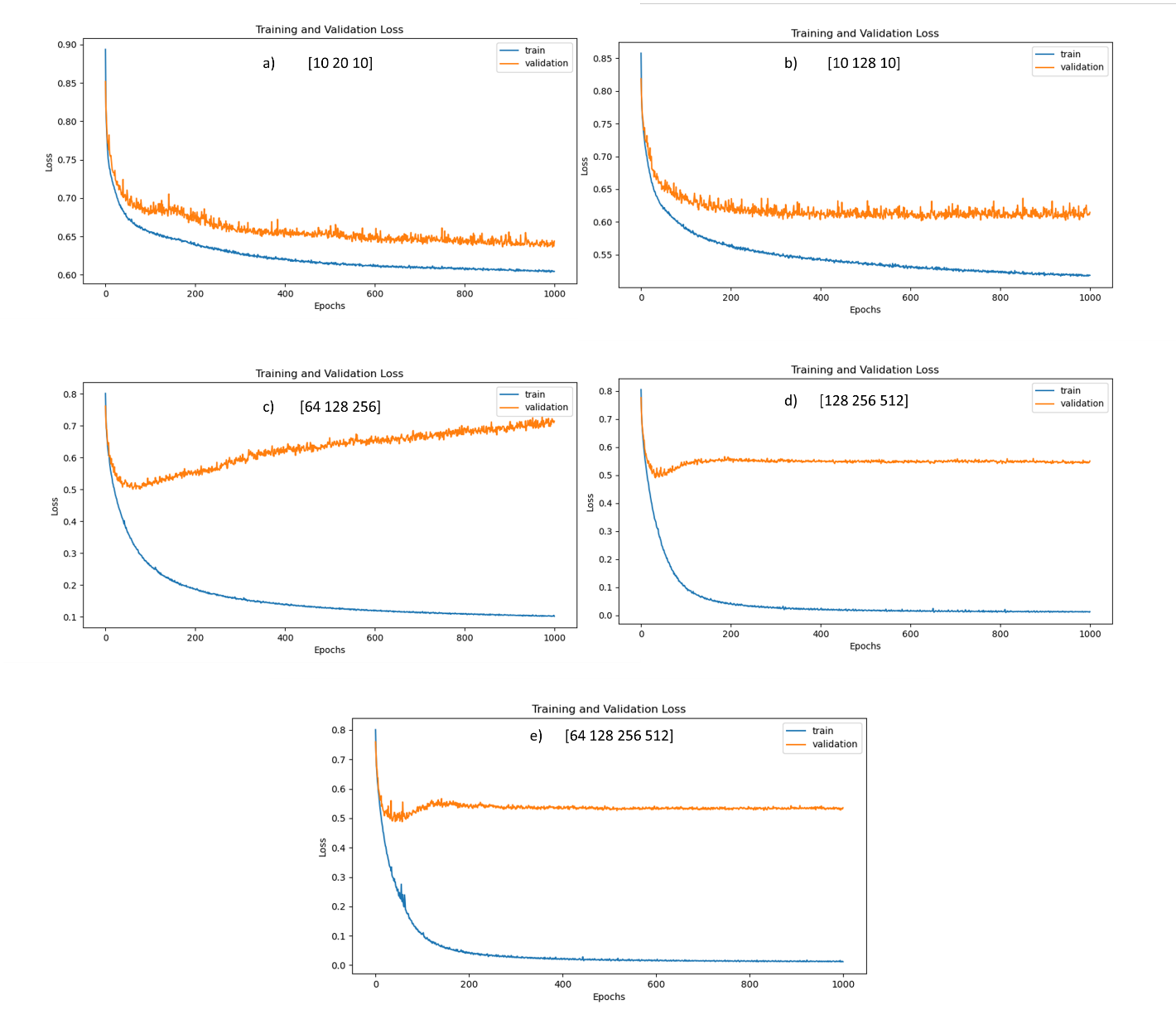


**Figure S.1**: *Illustration of the training and validation loss curves according to the trained networks by using different a different number of layers and neurons.*

With our chosen network architecture, we have tested the prediction of intracellular pH changes by (i) using only the CEST input data, summarizing the B0-corrected and normalized Z-spectra and MTRasym, and (ii) by additionally providing the quantitative measured T1 value of water. The results between ground-truth and prediction show a slightly improved RMSE as well as mean absolute value (MAE) and mean absolute percentage error (MAPE), when the quantitative T1 value is included as additional input parameter. This slightly better prediction due to the addition of quantitative T1 is also reflected visually by the generally lower pH value and the increased discrepancy between ground truth and prediction. However, we would like to emphasize again that such a model could in principle perform better with more CEST input data, and that the importance of T1 may play a minor role, but is still important in theory as CEST effects scale with R1 of water.


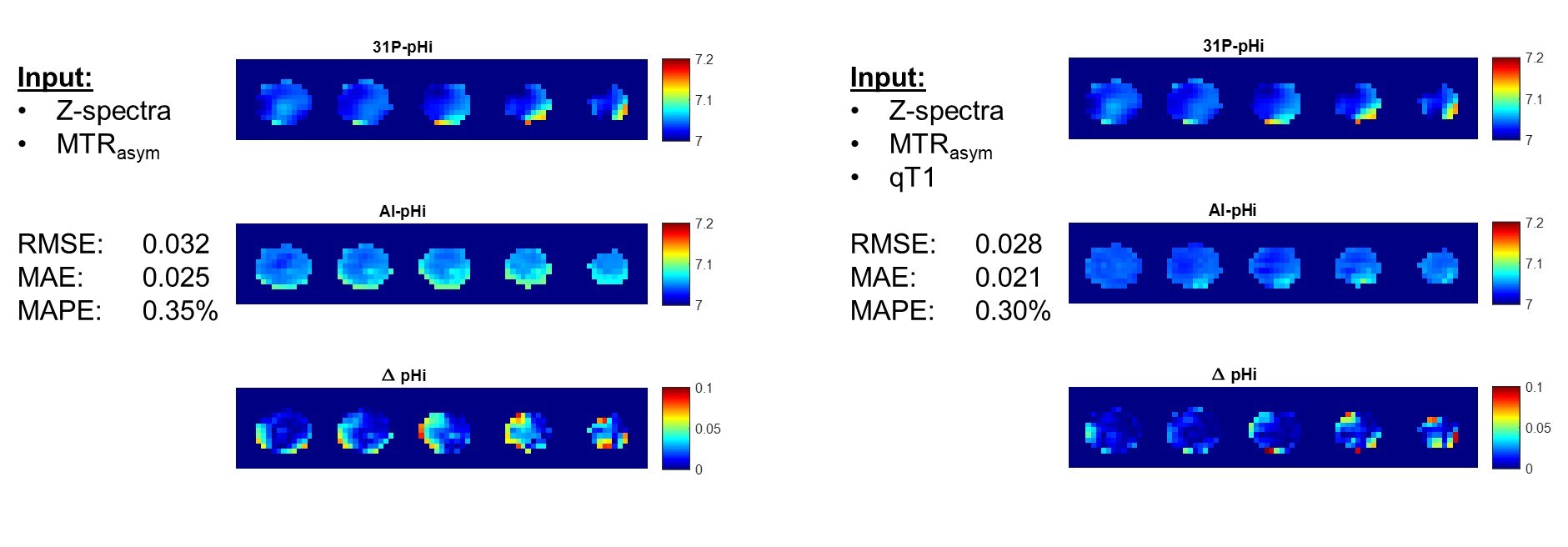


***Fig.S2****: Comparison of the model performance and the impact of T1 in addition to figure 5 by (i) providing the model just CEST data and (ii) with additional T1. The results reveal almost similar predictions but a slight improved correspondence when T1 is also addressed.*

The performance of the model also depends on the target data, which in our case were the calculated pHi values from the available spectra. We have included three different 31P spectra at the end, which were fitted in jMRUI using AMARES - a non-linear-least-squares (NLLS) quantitation algorithm. We show the fitted spectrum and residual signal after the fit for (a) the white matter (WM), (b) the border region between WM and GM, and (c) difference between WM and tumor tissue.

When observing the residual signal, it appears that fitting with AMARES gives a good result. However, it is well known that the intracellular pHi, which depends on the spectral distance between Pi and PCr, is elevated in tumor tissue. This can be also seen in (c). However, due to the bigger voxel size by 30x30x25 mm³, PVE from e.g. healthy white matter shift the signal of Pi to the right, leading in a reduced pHi value. Thus, the real pHi value in tumor could be higher. As a consequence, this issue impacts the prediction of our proposed model. In future this could be solved by addressing a higher spatial resolution of the phosphorus data.


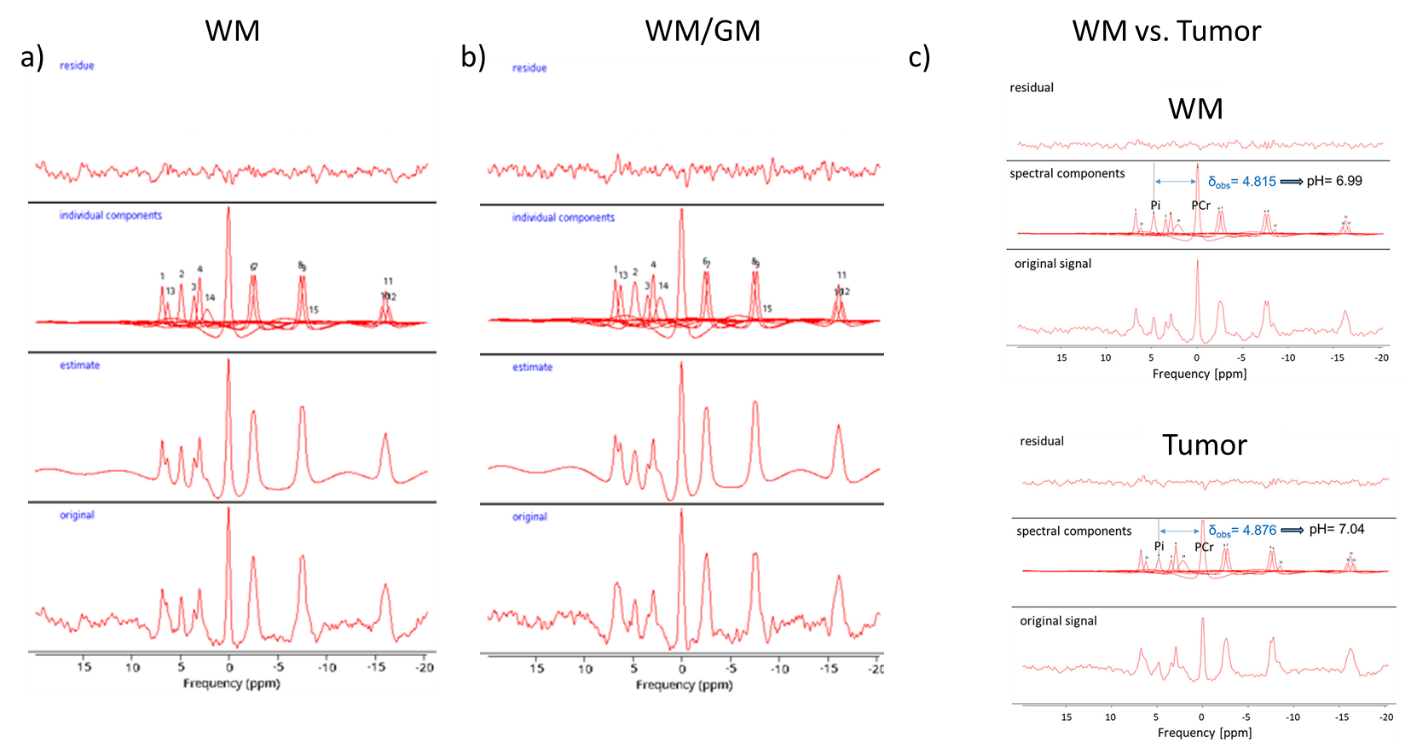


**Fig.S3:** Fitted ^31^P-spectra from the brain, acquired at 3T and post-processed with jMRUI and the embedded tool AMARES (Advanced Method for accurate, robust, and efficient spectral fitting). When observing the spectra in WM (a) and WM/GM boundaries (b), both spectra seem similar, indicating the impact of WM due to PVE. When comparing WM and tumor tissue, the ^31^P-spectra reveal a minor increase of the fitted spectral distance. However, in tumor tissue this might be also hampered due PVE.
